# Dynamic behavior modeling of laser-induced damage initiated by surface defects on KDP crystals under nanosecond laser irradiation

**DOI:** 10.1038/s41598-019-57300-2

**Published:** 2020-01-16

**Authors:** Hao Yang, Jian Cheng, Zhichao Liu, Qi Liu, Linjie Zhao, Jian Wang, Mingjun Chen

**Affiliations:** 1 State Key Laboratory of Robotics and System, Harbin Institute of Technology, Harbin, 150001 China; 2 Research Center of Laser Fusion, China Academy of Engineering Physics, Mianyang, 621900 China

**Keywords:** Mechanical engineering, Laser material processing

## Abstract

The issue of laser-induced damage of transparent dielectric optics has severely limited the development of high-power laser systems. Exploring the transient dynamic behaviors of laser damage on KDP surface by developing multi-physics coupling dynamics model is an important way to reveal the mechanism of nanosecond laser damage. In this work, KDP crystals are taken as an example to explore the mechanism of laser-induced surface damage. Based on the theories of electromagnetic field, heat conduction and fluid dynamics, a multi-physics coupling dynamics model is established for describing the evolution of nanosecond damage processes. The dynamics of laser energy transmission, thermal field distribution and damage morphology during nanosecond laser irradiation are simulated with this model. It is found that the enhancement of light intensity caused by surface defect plays an important role in the initial energy deposition and damage initiation of the laser irradiation area. The evolution of temperature field and crater morphology during subsequent laser irradiation is helpful to understand the laser damage process. The feasibility of this model is verified by the morphology information of typical defect-induced laser damage. This work provides further insights in explaining the laser-induced damage by surface defects on KDP crystals. The model can be also applied to investigate the laser damage mechanisms of other transparent dielectric optics.

## Introduction

Inertial confinement fusion (ICF) driven by high power lasers is a promising strategy for new clean energy^1–3^. Potassium dihydrogen phosphate (KDP) crystals are generally used as electro-optic switches and frequency conversion elements in ICF laser facilities, due to their specific physical properties, e.g., the wide-region spectrum transparency, relatively high nonlinear conversion efficiency and reproducible growth to the large sizes needed^2,4^. However, tiny defects (e.g., pits, scratches and cracks) are inevitably generated on the KDP surfaces during the processes of ultra-precision machining due to its weak mechanical properties. Structural defects on the surface of optical component would reduce their own laser damage resistance^5,6^. With the required increase of output laser power, the laser-induced damage (LID) of crystal components has become a major issue, limiting the development of ICF laser systems.

In recent decades, laser-induced damage of optical components has been a hot topic around the word^7–9^. At present, LID models for transparent dielectric optics can be generally classified into avalanche ionization models, electromagnetic field models, thermal models, and hydrodynamic models. Jupé *et al*.^10^ solved the laser-induced damage threshold in optical component using the ionization models combining with photo-ionization processes, avalanche ionization processes and relaxation processes from conduction band to other electronic states. The contribution of ionization phenomena was assessed during the ultra-short laser pulse and they found that the laser-induced damage threshold was dependent on the laser wavelength. Chen *et al*.^11^ simulated the light field distribution by calculating the electromagnetic field equation and found that some cracks with specific shapes and dimensions on KDP surface can produce light intensification as high as hundreds of times, which is sufficient to initiate the laser-induced damage. With modeling and experimental methods, Carr *et al*.^12^ studied the behavior of solid-state laser supported absorption fronts generated in bulk fused silica under high-power nanosecond laser pulses. They found that laser damage size was dependent on laser intensity and mainly driven by the temperature-activated optical absorption. DeMange *et al*.^13^ used the experimental and hydrodynamic modeling to investigate the early-period laser damage dynamics to explain the mechanism of bulk laser damage during nanosecond laser irradiation. The results suggested that the hydrodynamic instability at the unstable phase-changing interface between the two phases in the damage region played a major role in generating the cracks in the solid optics of high stress.

The main mechanisms of laser-induced damage of femtosecond pulse lasers and nanosecond pulse lasers are intrinsic ionization and defect-based heat absorption, respectively^14–17^. Because the duration of nanosecond laser damage is longer, multiple physical mechanisms like light modulation, heat absorption, fluid flow, etc. are included and even coupled with each other during the damage processes^18–20^. Previous work on laser damage has researched on the effect of pulse duration or wavelength on bulk damage, the mechanism of impurity-induced “explosion” inside the boules and the light modulation of surface cracks. However, the calculation of a single thermal or electromagnetic field model could not explain the laser damage mechanism well. In order to comprehensively simulate the damage process during nanosecond pulses, different physical mechanisms need to be considered. Besides, previous studies on laser damage for optical elements mainly focused more on bulk damage, which is not as complex as surface damage due to the changeable surface defects and the participation of air during damage initiation. Non-isothermal fluid action caused by the interaction between the thermal field and the flow field is very violent. Therefore, the mechanism of LID on KDP surface under nanosecond laser irradiation is still not clear. With the continuous improvement of KDP crystal growth technique and laser conditioning process, the bulk damage of KDP crystal exposed with strong laser irradiation (high electric field) has been well restrained^21,22^. However, surface damage is still a major issue, limiting the enhancement of damage threshold due to the existence of manufacturing-induced defects. In a word, it is of great theoretical and practical significance to explore the laser-induced surface damage mechanisms, which are beneficial to optimize the optical manufacturing processes and develop the laser damage resistance of KDP crystal.

Fortunately, the multi-physics coupling technology provides an access to study the mechanism of laser damage induced by machining defects on KDP surfaces under nanosecond laser irradiation. In this work, a multi-physics coupling model is firstly established to model the laser damage behaviors of KDP crystals under strong nanosecond lasers. Then, the evolution of damage processes such as laser propagation, heat deposition and crater formation is simulated. Further, a combination of micro-indentation and laser damage test is presented to experimentally verify the triggering effect of surface cracks on the laser damage processes of boiling and flowing. The novelty of this work is building a multi-physics model, coupling the electromagnetic field, thermodynamic and hydrodynamic fields. Using this model, the damage evolution from laser irradiation to crater formation is investigated to explore the mechanisms of damage initiation and its growth. The contribution of surface defect, energy deposition, phase transition and fluid action can be proved by the theoretical and experimental evidences. The results help us explore the generation mechanism of laser damage on KDP surface under nanosecond laser irradiation and provide new insights in understanding the effect of temperature and fluid on the damage evolution, which are essential for optimizing the surface processing technic and improving the optics laser damage resistance.

## Model and Theory

**Figure 1:**
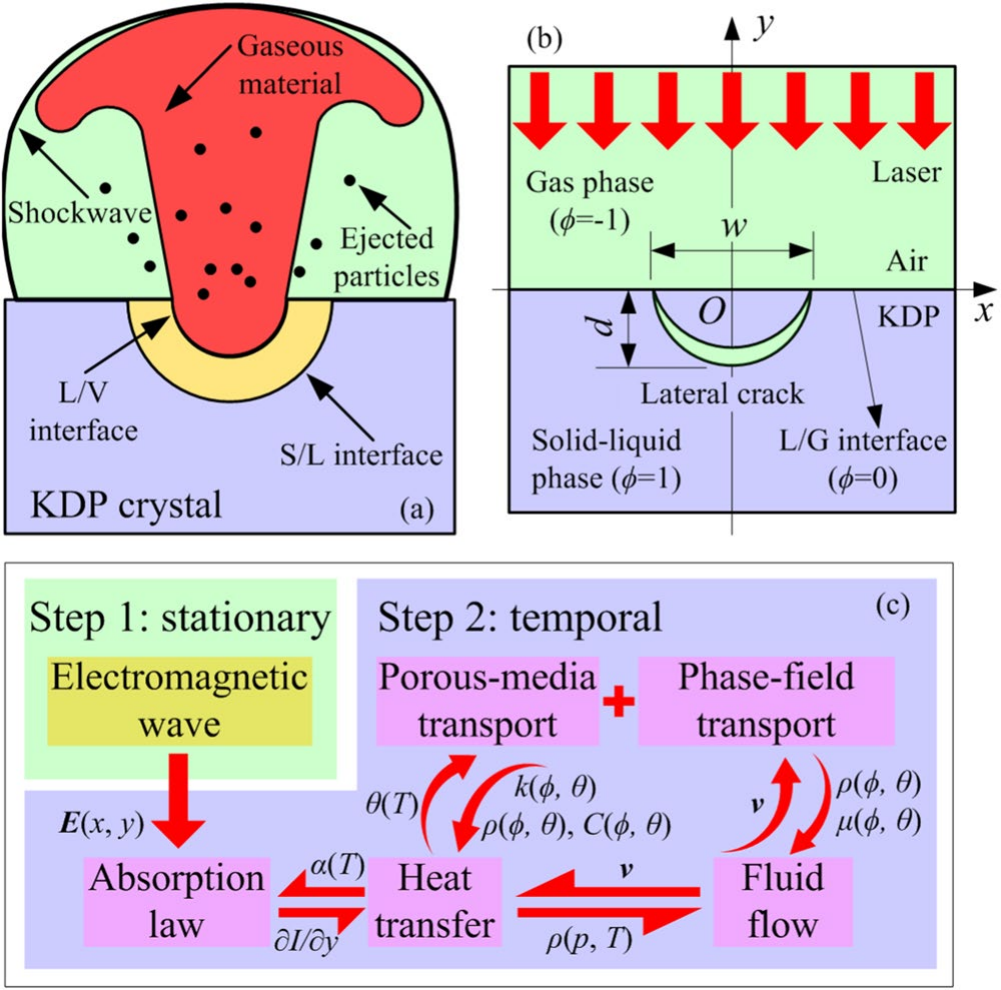
LID model initiated by surface defects on KDP crystals under nanosecond lasers. (**a**) Diagram of local damage region of KDP crystal under the nanosecond laser. (**b**) Calculation schematic of multi-physics coupling model for LID model initiated by lateral crack on KDP crystal under nanosecond laser irradiation. (**c**) Calculation flow chart of the multi-physics coupling model.

The surface damage processes of KDP crystals under nanosecond laser irradiation are complicated. Figure 1(a) is the diagram of local damage region of KDP crystal under the nanosecond laser irradiation. The local area of damage can be regarded as a mixed state of solid, liquid and gas. During the damage processes, sharp changes of physical parameters such as local temperature or pressure produce the strong shock wave around the damaged area. Besides, the ejection phenomenon would also occur during the damage processes due to the phase change and fracture of substrate material. The initiation of laser damage can be attributed to the existence of surface defects. The structural defects are introduced on the KDP surface during the manufacturing process^11,23^. Under the strong laser irradiation, the light intensity inside the KDP crystal is enhanced due to the special geometric structure of the defect. Then there will be a local light intensity hot spot near the defect, which induces the generation of free electrons in the insulator by ionization^24^. Thus, an electromagnetic field model is used to calculate the laser energy propagation characteristics in crystal during laser irradiation. Under the subsequent laser irradiation, free electrons interact with the laser and then plasma is generated, which makes the temperature of the material increase rapidly due to the inverse bremsstrahlung. The increasing temperature of the material will in turn promote the production of free electrons inside the crystal. A re-write energy equation needs to be modeled at this moment. When the material temperature rises to the melting point or boiling point of the crystal, dynamic behaviors such as melting, boiling and even material flow would occur in the local damaged area. In addition, the evolution of local mechanical properties during the phase changes^25^ is also an important cause of crystal surface damage, especially near the defects with low mechanical strength. Therefore, the physical equation describing the material phase change and fluid flow is indispensable in the model.

Figure 1(b) is the calculation schematic of multi-physics coupling model for LID model initiated by lateral crack on KDP crystal under nanosecond laser irradiation. The total calculated domain is a rectangle with width of 4 μm and height of 5 μm. The model is divided into two parts: the gas phase domain and the solid-liquid phase domain. On the surface of the solid KDP, there is an initial lateral crack with width of 600 nm and depth of 300 nm. The incident light propagates along the -*y* direction perpendicular to the front surface of the crystal.

The calculation flow chart of multi-physics coupling model is shown as Fig. 1(c). The calculation can be mainly divided into the first-step solution for frequency domain steady state and the second-step solution for time domain evolution to obtain the characteristics of laser propagation, energy deposition and phase change. The calculation process of the whole model is based on the finite element method (FEM) and the multi-physics coupling algorithm. The algorithm of multi-physics coupling calculation is mainly based on the technology of constructing simultaneous equations including different physics governing equations to directly assemble multiple partial differential equations into a total stiffness matrix, and simultaneously solve different physical parameters in the equation set. With the great improvement of the computer’s solving ability, it is possible to solve nonlinear complex systems with physical parameters coupled to each other.

Firstly, the Maxwell equations are used as governing equation to solve the electromagnetic wave propagation characteristics during the laser action^15,26^. Due to the fact that the surface defect of KDP crystal in practical engineering is far smaller than the spot size of internal optical path in large laser system, we assume that the incident light is a harmonic plane electromagnetic wave and the equations can be simplified to the Helmholtz equation, as Eq. (1). Based on the light intensity distribution ***E***(*x*, *y*) near the lateral crack on the front KDP surface, the temperature of the hot spot solved by the electromagnetic field model is assumed to be 9000 K at a the initial time due to the breakdown effect. Then, energy deposition at different moments under laser irradiation is obtained by solving the absorption law between laser and material. The Beer-Lambert law is employed to describe the energy absorption of the incident laser by the material^12^. For a beam of incident light intensity with *I*_laser_, this law can be written as the differential form, as Eq. (2). The algorithm for solving the thermal distribution inside the crystal is mainly based on the governing equation of heat transfer, as Eq. (3). And the incompressible Navier-Stokes Eq. (4) is chosen to describe the velocity in these domains^27^. The governing equations set in the multi-physics coupling model for laser damage can be described as follows:

1 $$\nabla \times (\nabla \times {\boldsymbol{E}})-{k}^{2}{\varepsilon }_{{\rm{r}}}{\boldsymbol{E}}=0$$

2 $$\frac{\partial {I}_{{\rm{laser}}}}{\partial y}=\alpha (T){I}_{{\rm{laser}}}$$

3 $$\rho C[\frac{\partial T}{\partial t}+\nabla \cdot ({\boldsymbol{v}}T)]=\nabla \cdot (\kappa \nabla T)+{I}_{{\rm{laser}}}\alpha (T)-\frac{\Delta L\dot{m}\delta (\varphi )}{M}$$

4 $$\rho [\frac{\partial {\boldsymbol{v}}}{\partial t}+{\boldsymbol{v}}\cdot (\nabla \cdot {\boldsymbol{v}})]=\nabla \cdot [\,-\,p{\boldsymbol{I}}+\mu (\nabla {\boldsymbol{v}}+{(\nabla {\boldsymbol{v}})}^{T})]+{{\boldsymbol{F}}}_{{\rm{Darcy}}}$$

Here ∇ is the differential operator, ***E*** is the electric field intensity, *k* is the wave number and *ε*_r_ denotes the relative dielectric constant. *y* is the coordinate along the propagation direction of the laser beam, *T* is temperature of each point in the solution domain and *α*(*T*) is temperature-dependent heat absorption coefficient. *ρ*, *C* and *κ* are the density, heat capacity and thermal conductivity of KDP crystal, respectively, in different conditions^19,28^. Δ*L* is the latent heat during the phase change. $\dot{m}$ is the evaporation rate on the liquid-gas interface. *δ(φ*) represents the Dirac delta function. *φ* is the solution variable of phase-field equation. ***v*** is the velocity vector at each point in the solution domain. ***I*** is the unit matrix, and *μ* is the dynamic viscosity of liquid phase for KDP crystal. *p* is the local hydrodynamic pressure. The Darcy friction ***F***_Darcy_ on the solid-liquid interfaces represents the lost momentum of the solid and liquid phases.

The 3*ω* (*λ* = 355 nm) plane wave along the direction normal to the front KDP surface was adopted in this calculation domain. The meshing size in the FEM calculation is less than one-sixth of the laser wavelength. The incident laser is TE polarization mode with light intensity *I*_laser_ = 10 GW/cm^2^ and flat-top pulse width of 4 ns. The light intensity enhancement factor (LIEF) is used to describe the local light intensification relative to perfect crystal without any defects on the surface. Other model sets of the electromagnetic field parameters can be referred to ref. ^29^.

The heat absorption coefficient of KDP crystal under high temperature conditions is obtained by re-writing. Saito has introduced a method based on the vibrational band edge distortion to solve heat absorption coefficient. It is pointed out that the absorption coefficient model is widely used for crystalline solids, because deviation from perfectly ordered systems can also be generated by the thermal vibration inside crystals^30^. Since heat absorption coefficient of the crystal at room temperature is very low (0.005 cm^−1^), the re-write law needs to satisfy that the absorption coefficient must have sufficient response when the crystal temperature reaches 2273 K, which is the temperature of phase change under a high pressure condition^19^. Besides, when the temperature is high enough, the heat absorption coefficient would keep stable at a higher value.

Since the two-dimensional model is built in this work, the optical anisotropy of the component is not considered in the calculation process. Besides, the effect of material phase change on the calculation results of the model is sufficient to replace the effect of its parameters with temperature variation. Thus, the precise influence of temperature on material parameters can be temporarily ignored.

The energy absorption module and the heat transfer module can be bidirectionally coupled through local energy deposition and iteration of local temperature values. Finally, the positive feedback of the local temperature rise to the laser damage process during the interaction of the laser and material would achieve.

The setting of the fluid flow module is complex, mainly because the particles in different fluid media have various motion states. Even in the local microscopic regions of the material phase interface, there may be opposite forms of motion and huge velocity gradients. For the solid and liquid phases of KDP crystals, it is assumed that they are incompressible.

The material becomes boiling at the temperature above 3273 K^28^. When the temperature and pressure of the gas material change, its density will also change greatly. Thus, the Navier-Stokes equation for compressible flow is chosen as the governing equation in the gas domain.

The velocity of the liquid in the outward direction normal to the gas phase side is higher than that of the gas. Because there are three forces that act on the liquid on the interface, the natural boundary condition near the liquid-gas interface for the liquid domain is described by Eq. (5), which guarantees a force balance on the interface.

5 $${\boldsymbol{n}}\cdot [-{p}_{{\rm{l}}}{\boldsymbol{I}}+{\mu }_{{\rm{l}}}(\nabla {{\boldsymbol{v}}}_{{\rm{l}}}+{(\nabla {{\boldsymbol{v}}}_{{\rm{l}}})}^{T})]=\dot{m}({{\boldsymbol{v}}}_{{\rm{l}}}-{{\boldsymbol{v}}}_{{\rm{g}}})+\sigma {\boldsymbol{n}}\kappa \delta (\varphi )+{\boldsymbol{n}}\cdot [-{p}_{{\rm{g}}}{\boldsymbol{I}}+{\mu }_{{\rm{g}}}(\nabla {{\boldsymbol{v}}}_{{\rm{g}}}+{(\nabla {{\boldsymbol{v}}}_{{\rm{g}}})}^{T})]$$

Where *σ* is the surface tension coefficient, 0.0588 N/m. ***n*** is the liquid-gas (L/G) interface unit normal vector.

The continuity equations of hydrodynamics characterize the mass conservation in this system. But some transformations are employed for governing equations on various calculation domains.

The solid-liquid phase is incompressible in the model, And the continuity equation at the liquid-gas interface needs to be modified to represent the phase change from liquid to gas^31^.

The method of heat transfer in porous medium interface is utilized to describe the change from solid phases to liquid phases^32^. The temperature equation defined in porous media domains is based on the convection-diffusion theory with thermodynamic properties averaging models to describe both the solid matrix and fluid properties. The moving speed of the phase change material is dependent on the temperature of each calculation unit. When the moving speed reduces to an infinitesimal value, the porosity of the local domain is defined as zero, meaning that the material is completely solid. By solving the porous-media transport, a variable *θ* of solid-phase volume fraction that describes the solid-liquid state of the material would be obtained.

The L/G interface is tracked by phase-field transport methods^33^. The gas phase domain and the solid-liquid phase domain are represented by phase-field variable *ϕ* = −1 and *ϕ* = 1, respectively. Since the phase transition process is involved in the model, it is necessary to modify the traditional phase-field model and add terms related to phase transition. The algorithm is developed from statistical physics. Based on the Ginzburg-Landau phase change theory, the phase-field variable is obtained by the combination of differential equations like reaction diffusion, ordering potential and thermodynamic driving force. Then, the state of the material and the L/G interface in the solution domain can be determined according to the phase-field variable.

Coupling the physical government equations such as heat conduction, fluid flow, porous-medium transport and phase-field transport, we simulate the dynamic laser damage behavior and the evolution of typical physical parameters like temperature and phase-field variable. By establishing the laser-induced damage model initiated by KDP surface defects under laser irradiation, the dynamic behaviors of laser energy propagation, energy absorption, material change and fluid flow in the process of damage can be solved, which provides support for the exploration of damage mechanism.

## Experimental

**Figure 2:**
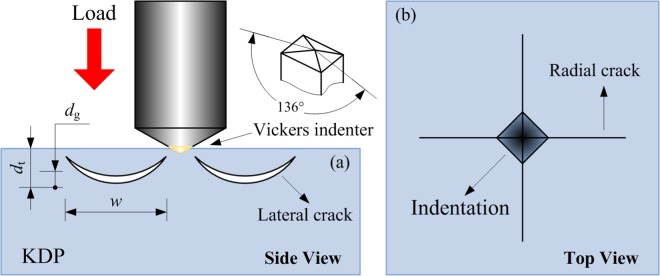
Schematic diagram of artificial cracks prepared by Vickers hardness tester. (**a**) Crescent lateral cracks generated on both sides of the indenter. (**b**) Peripheral radial cracks formation due to the existence of the cutting edge on the indenter.

In order to verify the effect of surface defects on LID of KDP crystal and the damage processes like boiling or melting, the experiments were performed by laser damage test on typical surface defects and observing the morphology of damage sites. Defects on KDP surface are common with various morphology and structure. This is because the KDP crystals are soft-brittle and sensitive to the temperature changes. Thus, some defects are inevitably introduced on the KDP surface from accidental vibration of the machine tool during daily machining, handling for cleaning, or even slight changes in external temperature. However, surface defects are generated randomly and there are no nearly identical defects that can be applied for repetitive experiments. Through statistical analysis for large amount fracture behavior of brittle materials, the microcracks on the manufactured surface or subsurface of brittle material (e.g. KDP crystals) are roughly divided into conical cracks, lateral cracks and radial cracks^18,34,35^. As for the lateral and radial cracks, they are mainly generated by a Vickers indenter on the substrate surface. Therefore, we choose artificial cracks prepared by Vickers hardness tester on KDP crystal surfaces to perform the laser damage test. Figure 2 is the schematic diagram of artificial cracks prepared by Vickers hardness indenter. The standard Vickers indenter with the section angle of 136° is adopted to apply a load of 100 g (0.98 N) on the surface of the crystal. For general isotropic optical elements, when their surface is squeezed by a sharp object similar to the Vickers indenter, crescent lateral cracks would be generated on both sides of the indenter, as shown in Fig. 2(a). In addition, due to the existence of the cutting edge on the indenter, radial cracks will be generated around the indentation, as shown in Fig. 2(b). This kind of radial crack is usually thin and long with less obvious effect on light scattering than that of lateral crack.

**Figure 3:**
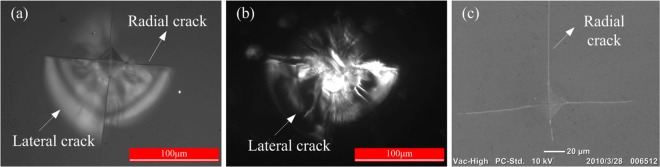
Indentations with peripheral cracks around on the crystal surface after loading: (**a**) the optical micrograph in bright field; (**b**) the optical micrograph in dark field; (**c**) the scanning electron micrograph.

The indentations with peripheral cracks around on the crystal surface after loading are presented in Fig. 3. Figure 3(a–c) are bright-field optical micrograph, dark-field optical micrograph, and scanning electron micrograph (SEM) of Vickers indentations and cracks around on KDP surface respectively. Comparing with different micrographs of the indentation pit, there are four obvious radial cracks and lateral cracks around the indentation. However, due to the anisotropy of the KDP crystal, the length of the radial cracks varies with the fracture direction. The upper radial crack is the longest, followed by the left and right cracks. The lower crack is the shortest. Furthermore, all of the lateral cracks are almost above the indentation.

**Figure 4:**
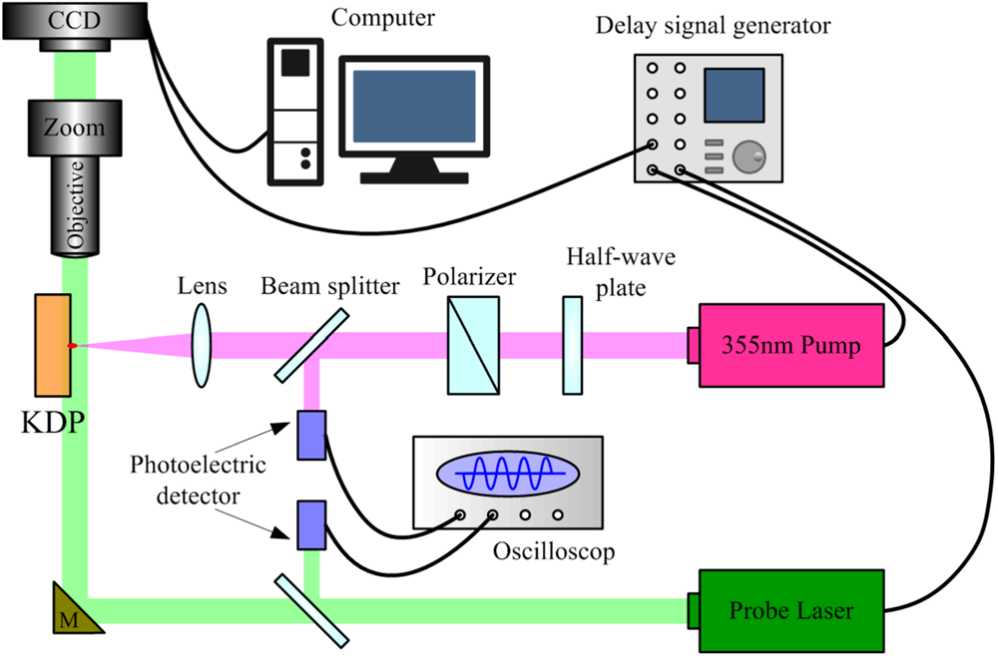
Diagram of light paths for generating the laser-induced damage sites on KDP crystal.

Figure 4 is the diagram of light path for laser damage test on KDP crystals. A strong pulse generated by Nd: YAG pump laser is transmitted by energy regulator, wave plate and focusing lens and eventually focused on the front surface (the laser incident surface) of KDP samples, where are located early prepared artificial cracks by Vickers hardness. The single-shot irradiation mode with a wavelength of 355 nm, pulse width of 8.3 ns (FWHM), and energy density of 38~41 J/cm^2^ was performed on the indentation pits. The output laser energy has a spatial distribution of Gaussian profile on the front surface of the crystal with the effective spot diameter of 388 μm (1/e), which is large enough to radiate the whole region of each defect. The change of surface morphology during each laser pulse irradiation is detected *in situ* using a CCD camera. In addition, according to our previous work, the light intensity enhancement generated by the slender radial cracks is much lower than that of the lateral cracks^11^. Therefore, we believe that the damage initiation is mainly caused by the lateral crack in this laser damage test. However, it can be seen from Fig. 3 that even the lateral cracks in a certain location are not a single crack. They are a cluster formed by numerous cracks with various shapes and sizes. Thus, the small difference between the lateral cracks would inevitably affect the damage results. But it also provides a good access to investigate the mechanism of LID initiated by the surface defects. As for the detection system for capturing the transient damage behavior, it consists of a probe laser, microscope imaging device and an electronic delay system. The probe pulse with 523 nm wavelength and ~70 ps duration (FWHM) is used for strobe-light illumination. The timing of the probe pulse in reference to the pump pulse is controlled by a delay signal generator. The resolution of relative time delay between pump and probe is <0.1 ns. The microscope is positioned perpendicular to the normal direction of the sample surface, to capture the process of transient behavior of the KDP crystal during laser damage. After the laser irradiation, the morphology of damage sites after each laser pulse irradiation is detected by scanning electron microscope to analyze the effect of surface defects on laser damage and the mechanism of damage under strong laser pulse.

## Results and Discussions

Results of physical parameters correspond to the whole equations set can be obtained by the multi-physics coupling model in a single solution. This section firstly shows the calculation of light field, thermal field and crater morphology during the nanosecond laser irradiation. Then the mechanism of laser damage on KDP surface during the strong laser irradiation is discussed. Next, the experimental results of laser damage test and damage morphological are analyzed to verify the dynamics process simulated with the coupling model of multiple physical fields.

**Figure 5:**
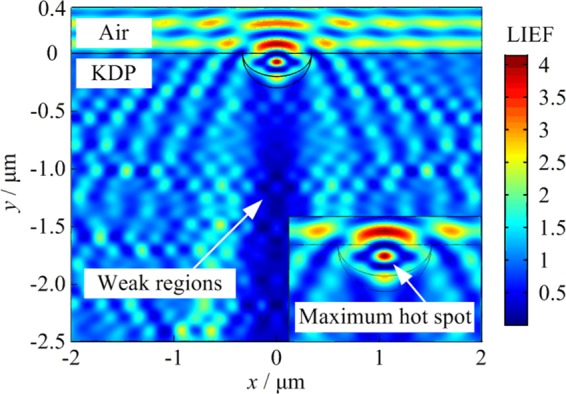
Distribution of light intensification near the lateral crack with the parameters of width *w* = 600 nm and depth *d* = 300 nm respectively. The insert represents the maximum light intensification of the electromagnetic-field simulation.

The simulation result of laser light propagation is shown in Fig. 5. The distribution of light intensification corresponds to the parameter LIEF. The LIEF can be calculated by |*E*/*E*_0_|^2^. Parameters *E* and *E*_0_ are the electric field intensity inside the KDP crystals with surface defects and ideal surface, respectively. When the lateral crack is located on the front KDP surface, light diffraction will occur around the defects inside the crystal due to the disturbed light propagation. In the cross-sectional profiles of LIEF along *xOy* plane of the Cartesian coordinates, some areas are much brighter, indicating that there is larger light intensity enhancement in these locations. However, some weak regions of light intensity are located behind the lateral crack. Uneven distribution of light intensity would directly result in differences in subsequent energy deposition. Besides, there is an elliptical hot spot in the center position of the lateral crack whose relative intensity is more than 4 times. However, local light intensification with value more than 4 is unacceptable generally^29^. In addition, the heat absorption coefficient will also change greatly at that time due to the sub-band gap level structure and the photosensitive contaminant in the vicinity of the defect. Thus, the local energy deposited by the hotspot of laser light provides the energy source for the subsequent damage process. As long as the initial deposition energy reaches a certain threshold, the subsequent damage processes, such as material thermal action and phase transition, can be initiated under the action of nanosecond laser. These dynamic processes would finally affect the eventual damage morphology of the crater.

**Figure 6:**
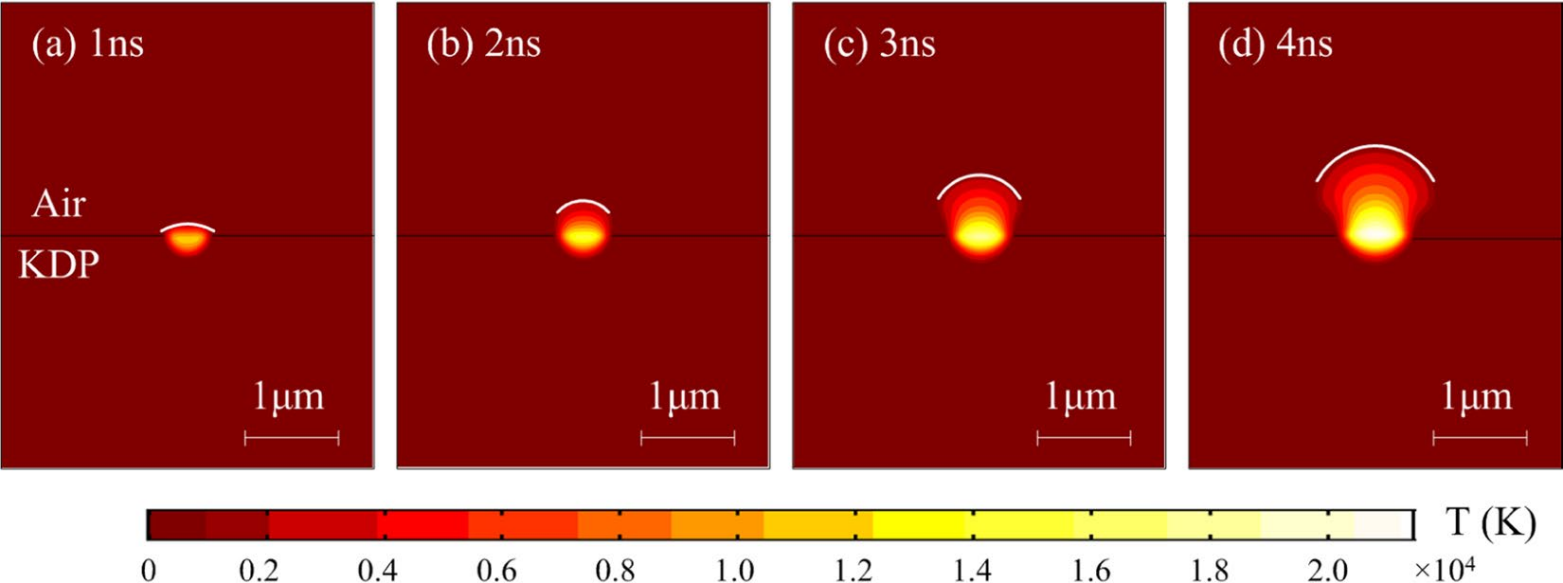
Variation of temperature distribution simulated by the model in the laser damage area at laser irradiation times of (**a**) 1 ns; (**b**) 2 ns; (3) 3 ns and (**d**) 4 ns, respectively.

Figure 6 is the variation of temperature (parameter *T* in the model) distribution simulated by the model in the damage area with time during nanosecond laser irradiation. With the continuous irradiation of the strong laser, the temperature in the damage site increases. The cross-section area of high-temperature region (HTR) gradually expands. Especially in the gas phase domain, the expansion rate of the HTR is particularly severe. At the irradiation time of 3 ns, the local temperature of the damage exceeds 10000 K, which is in good agreement with previously reported experimental results in ref. ^36^. The HTR is mainly composed of gaseous KDP, which forms plasma during the interaction between KDP and strong laser. Under the subsequent laser irradiation, it will absorb a large amount of laser energy and aggravate the damage behavior. Besides, it is generally accepted that physical quantities (e.g., temperature, density or pressure) with tremendous changes would produce shock waves on the step surface (the surface consists of many points in space at which values vary widely) of the corresponding physical parameters. In the temperature simulations, the step surface of high temperature in the damage site is represented by white lines, whose changes are consistent with that of plasma interface previously reported in ref. ^18^. When KDP crystal is exposed with high power nanosecond lasers, the interaction between laser and matter produces a large amount of energy deposition. Then the vapor produced in the initial stage rapidly transforms into plasma under the action of high temperature environment and strong electromagnetic field. The deposition energy in the damage area provides necessary conditions for subsequent plasma expansion.

**Figure 7:**
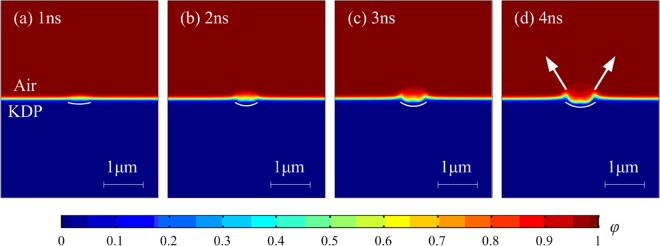
Laser damage crater morphology simulated by the model in the laser damage area at laser action time of (**a**) 1 ns; (**b**) 2 ns; (3) 3 ns and (**d**) 4 ns, respectively.

The phase-field variable *ϕ* calculated in the multi-physics coupling model could represent the laser damage crater morphology. Figure 7 is the variation of crater morphology simulated by the model in the laser damage area with time during nanosecond laser irradiation. When the laser acts for 2 ns, the laser damage pit begins to appear. When the laser acts for 3 ns, the boundary of laser damage site gradually becomes obvious. In the early stage of laser damage, the lateral expansion speed of the damage site is larger than that in the depth direction. The shape of the damage crater is like a bowl, and the depth-width ratio of the bowl becomes larger and larger with the increase of laser time. Moreover, the contour of the crater is similar to that of the high temperature area. Hence, it is inferred that the crater is mainly caused by the phase transformation of high-temperature materials generated by high power laser irradiation. As the laser action time increases, the fluidity of the material in the laser damage site increases. Due to the uneven distribution of fluid heat, local Marangoni convection is formed. The material moves rapidly to both sides of the crater. This phenomenon is consistent with the liquid ejection behavior experimentally observed in the laser damage processes reported in refs. ^37,38^. As the material properties of high-temperature phase transition region are affected by multiple physical parameters such as temperature, pressure, ionization and so on, a small parameter change would incur a huge impact on the morphology of laser damage crater. Thus, there are some slight fluctuations on the bottom of the crater because of the instability of the damage system.

**Figure 8:**
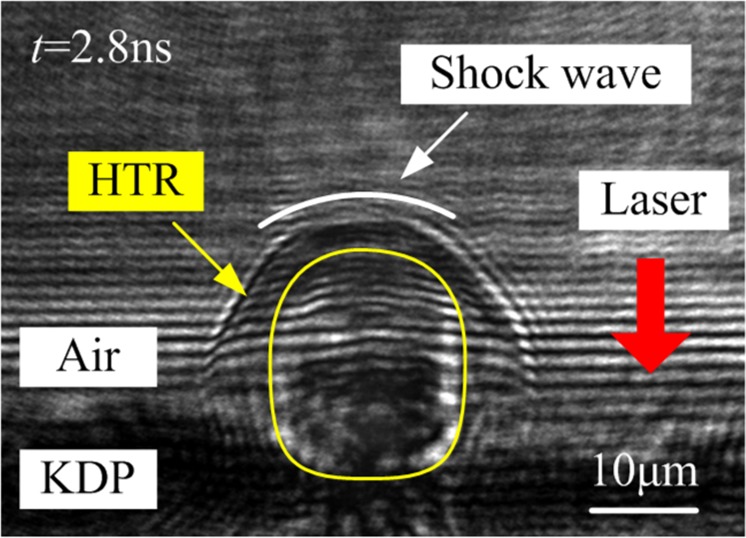
Transient dynamic behavior at early stage (*t* = 2.8 ns) of the laser-induced damage initiated by surface defects on KDP crystals under nanosecond laser irradiation.

Based on the time resolved pump and probe method, the early kinetics of laser damage on KDP surface is captured. Figure 8 is the transient dynamic behavior of the laser-induced damage initiated by surface defects on KDP crystals under nanosecond laser irradiation. The laser is incident from the air side to the crystal surface. At the delay time *t* = 2.8 ns, a substance with different refractive indexes from the surrounding medium appears in the center of the damage, which can be seen inside the yellow curve in Fig. 8. Near the top of the yellow curve, there is a clear shock wave front (the white curve). It is generally believed that the shock wave appears at a position where the spatial variation of the physical parameter value is relatively drastic. Thus, the material wrapped by the middle yellow curve is inferred to be a high-temperature mass of gaseous KDP generated by laser damage on crystal surface. When the surface laser damage occurs, the local material of KDP crystal first undergoes a phase change, which generates a large amount of high-temperature gaseous substances. The existence of HTR intensifies the subsequent laser-material interaction. Although the transient temperature of the local laser damaged area cannot be detected directly by experiments, the similarity between the morphology of the HTR and the simulation results proves that the simulation results are correct. The HTR expands faster in the direction along the laser incident than in the vertical direction. Simulation results of HTR also share the similar rules. Besides, the color of the transient image in the bottom of the HTR is darker, which is due to the strong absorption from laser energy. As for the simulation results in Fig. 6, the highest temperature is indeed located at the bottom of the HTR. Thus, the simulation results are consistent with the experimental results.

**Figure 9:**
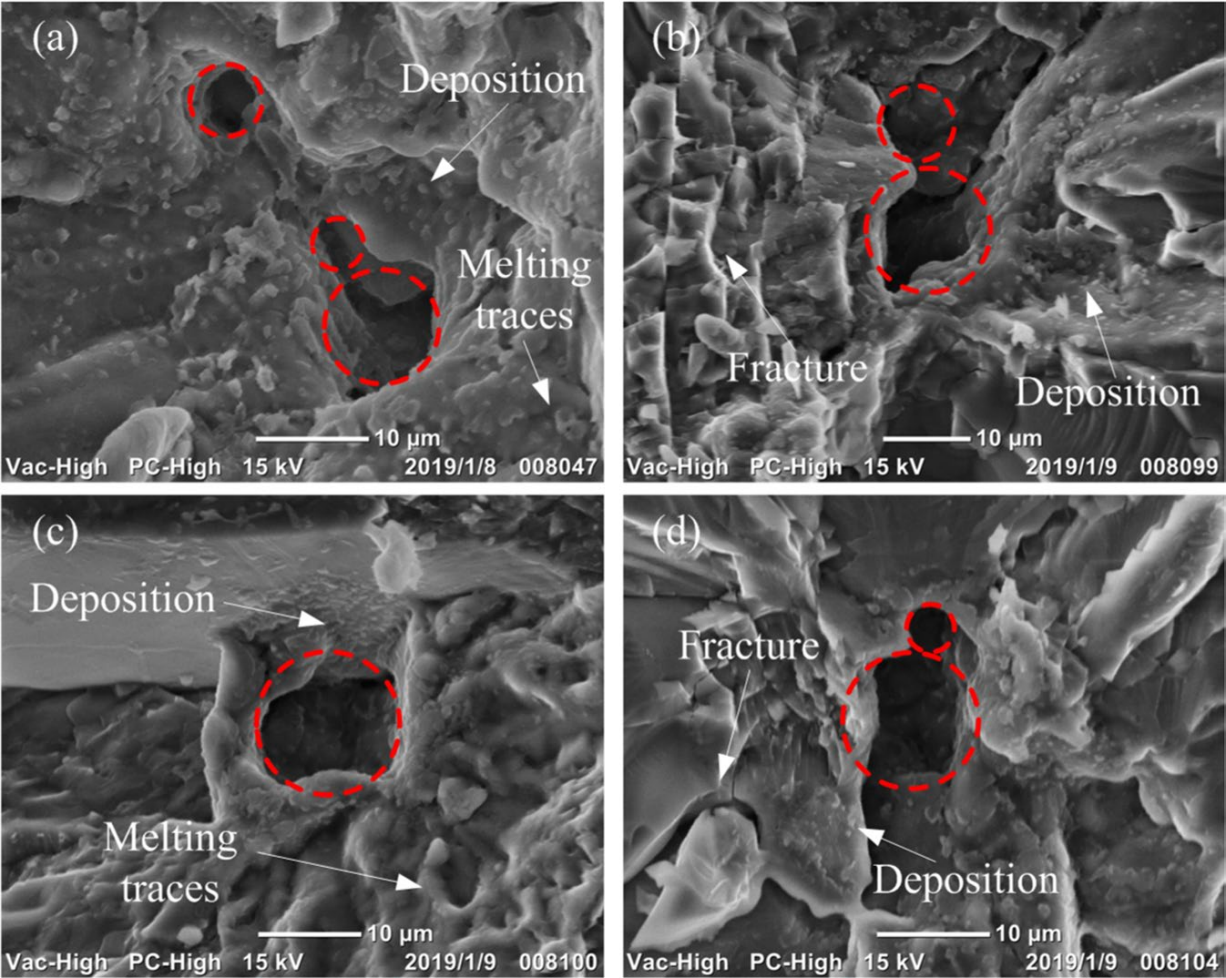
SEM of the laser damage site initiated by lateral cracks on front KDP surface under 355 nm wavelength laser irradiation. There are four different images of damage sites with the boiling cores located on the districts of original lateral cracks, (**a–d**), respectively.

The SEM of laser damage morphology after 355 nm-wavelength single-shot laser irradiation is shown in Fig. 9. Although the microstructure of the four damage sites is complex and changeable, boiling core (red circle in these images) and vapor deposition are their common characteristics. Figure 9(a,c) also display significant melting traces near the boiling core. Besides, neat and smooth fracture surfaces are around the boiling core instead in Figs. 8(d) and 9(b), where the vapor deposition can be also clearly seen. We deduce that during the damage process, the lateral crack leads to a large amount of energy deposition near the front surface of the crystal during the pulse laser irradiation. Then, series of hydrodynamic processes like melting, boiling and flowing are generated. The fracture is accompanied by a hydrodynamic process or occurs afterwards. The generation of fractures is mainly related to the temperature directly dependent thermal stress^39^, the temperature indirectly dependent hydrodynamic pressure, the expansion pressure produced by phase change, and the local mechanical properties of materials which are not dependent on the temperature. Therefore, if the laser energy deposition during the initiation of the damage is defined as the pre-damage process, the fracture may occur at any stage in the middle and late stages of the damage. When the fracture occurs in the melting process, the local damage region is still in a significant high temperature environment, and traces of melting can be found on the fracture surface at the final damage morphology. When the fracture occurs after the melting process, the damaged surface with the molten material will break away from the substrate as the material fracture, leaving a new smooth fracture surface. However, due to the relatively slow deposition process of gas phase materials, a large amount of vapor deposition can still be seen on some newly generated fracture surfaces. Under certain special conditions, the local defect even produces secondary fractures, which are mainly related to the local mechanical properties of the material. For example, assuming that the original site in Fig. 9(d) is under greater laser irradiation, the new crack surface may continue to expand under subsequent thermal shock until it completely separates from the substrate even if it is in the same damage process. Evidence of the effectiveness of the simulation can also be obtained from the damage morphology. Firstly, the boiling core acts as damage triggering point, which proves the significance of electromagnetic field simulation. And there is more than one initial damage point in some damaged sites, which are mainly related to the existence of lateral crack clusters before damage. Besides, obvious vapor deposition and melting traces can be seen around the core, which proves the involvement of thermal action and fluid action during the damage process. Finally, the circular shape of the boiling core is similar to the simulated bowl-shaped crater as shown in Figs. 6 and 7. In a word, the consistency between the experimental results and the simulation results proves the validity of the model.

Combined with the above simulation analysis, the original damage process can be understood as a sharp light absorption point on the surface or subsurface of the KDP crystal induced by manufacturing-induced defects. Then, the surrounding material interacts with the high power laser. The temperature rise of the material is accompanied with the melting, boiling, flowing, and even ejecting processes. The uneven distribution of the thermal field would cause a large thermal stress. Besides, fluid flow and phase change expansion also produce enormous recoil pressure. Once there is a weak point inside the crystal, the material will be broken and an obvious damage pit would be produced.

## Conclusion

Based on the theories of electromagnetic field, thermodynamics and hydrodynamics, a multi-physics coupling dynamics model is established to describe the behavior of laser damage processes. The propagation characteristics of laser energy, the evolution of temperature distribution and crater morphology in local damage area were simulated. Light intensifications modulated by lateral cracks lead to energy hotspots, which are sufficient to launch avalanche ionization and trigger laser damage on KDP surface. The high-temperature region formed by energy deposition plays an important role in the damage evolution process. It provides a power source for materials phase change, fluid flow and crater morphology evolution. Besides, plasma generated by high-temperature vapor interacts with the subsequent laser irradiation, which accelerates the laser energy deposition. The crater of laser damage presents a bowl-shaped morphology during the laser irradiation. As the increase of laser action time, there would be liquid ejecting around crater, which is mainly due to the uneven distribution of fluid temperature in the damaged area. The temperature of local damage area and the surface morphology of the shock wave obtained by simulation are consistent with the experimental results reported in the literature. By conducting laser damage test and observing laser damage morphology on KDP surface, it is found that lateral cracks are the dominant source to trigger the laser damage on KDP crystal. The experimental evidence of material boiling and melting on the damage sites is consistent with the simulation results. The detection of fracture surface also provides a new idea for the subsequent study of damage formation process by analyzing the evolution of stress and pressure. This work proves that the laser damage involves physical law of light, heat, fluid and fracture. Only when the multiple physics are considered at the same time can the laser damage mechanism be comprehensively explored. The dynamic model of LID initiated by surface defects under nanosecond laser irradiation can be expected to provide new insights into understanding the mechanisms of LID on manufactured KDP surface and optimizing the processing technic of high-quality KDP surface for improving its laser damage resistance.
